# Adolescents’ Internet addiction: Does it all begin with their environment?

**DOI:** 10.1186/s13034-023-00626-7

**Published:** 2023-07-04

**Authors:** Khansa Chemnad, Maryam Aziz, Azza O. Abdelmoneium, Sanaa Al-Harahsheh, Ahmed Baghdady, Fatima Y. Al Motawaa, Diana Alsayed Hassan, Raian Ali

**Affiliations:** 1 College of Science and Engineering, Hamad Bin Khalifa University, Doha, Qatar; 2 Doha International Family Institute, Qatar Foundation, Doha, Qatar; 3 World Innovation Summit for Health, Qatar Foundation, Doha, Qatar; 4 World Innovation Summit for Education, Qatar Foundation, Doha, Qatar; 5 Department of Public Health, College of Health Sciences, QU Health, Qatar University, Doha, Qatar

**Keywords:** Adolescent internet addiction, Family environment, School environment, Internet addiction, Academic performance

## Abstract

**Background:**

This is one of the few studies that examines adolescent Internet addiction (IA) among Middle Eastern population. The purpose of this study is to determine whether adolescents’ family and school environments play a role in their Internet Addiction.

**Methods:**

We conduced a survey that included 479 adolescents in Qatar. The survey collected demographic data, the Internet Addiction Diagnostic Questionnaire (IADQ), the Brief Family Relationship Scale (BFRS) and questions from the WHO Health Behavior in School-aged Children (HBSC) survey that assess school environment, academic performance, teacher support, and peer support of the adolescents. Factorial analysis, multiple regression, and logistic regression were used for statistical analysis.

**Results:**

Family environment and school environment were negative and significant predictors of adolescent Internet addiction. The prevalence rate was 29.64%.

**Conclusion:**

Results imply that interventions and digital parenting programs should not only target adolescents, but also include entities in the developmental environment of adolescents, i.e. their family and school.

## Background

People’s dependency on the Internet has increased over the past years, and it has been found that adolescents are the most adept at adopting and adapting to the Internet compared to other age groups [1]. A study in the US reported that adolescents felt they were spending the right amount of time on social media and found it hard to give up on their usage [2]. With the increased use of social media platforms such as TikTok, Instagram, and Snapchat among adolescents, there is a risk of addiction to the Internet. Internet Addiction (IA) also known as “problematic Internet use”, “compulsive Internet use”, and “pathological Internet use” is defined as the excessive or uncontrollable use of the Internet leading to symptoms of withdrawal and tolerance [3]. Although Internet Addiction has not been formally recognized as an actual diagnosis, increasing research has shown that IA is becoming a problem that may require professional treatment [4–6]. Over the past few years, several studies have been conducted on factors affecting adolescent Internet addiction. According to the literature, many negative mental health issues such as depression, anxiety, psychoticism, and somatization are associated with IA [7].

Adolescence is the period of physical, intellectual, emotional, and social change from childhood to adulthood [8]. During this period of development, adolescents are greatly influenced by various factors both internal and external to their social environments [9]. External factors such as changes in economic status or peer pressure can impact the decisions made by adolescents during this phase [10]. Internal factors include the developmental needs of an adolescent that are influenced by their interactions with their parents and other significant adults in their lives for example teachers [11]. The impact of these internal and external factors is seen in the patterns of adolescent well-being and behavior exhibited by the adolescent during this period [11]. Adolescence is a critical stage in the development of an individual, as they begin to explore their identity and become more independent from their parents [12, 13]. During this phase, adolescents experience conflicting emotions, such as excitement at gaining independence coupled with uncertainty and fear about the future [14]. These complex emotions result from a combination of biological and psychological changes that prepare them for adulthood [12]. Along with these changes, there is also a shift in psychological perspective as the adolescent views the world from a cognitive standpoint rather than from a more concrete perspective of childhood [15]. Adolescents are more likely to be at risk of mental health disorders as well as becoming addicted to various substances due to the increased levels of impulsivity and cognitive development occurring during this period [16]. We hypothesize that this may be the case for adolescent Internet addiction as well.

Based on Bronfenbrenner’s socioecological model, a developing individual, and their behaviors are influenced by various levels of ecological systems, including the immediate and direct environments they participate in (microsystem), the connections and interactions between these environments (mesosystem), the social settings that indirectly impact them, and the broader cultural and societal contexts [17]. The model offers insight into how the behaviors of an individual continue to change as the individual evolves as a result of the continuous interactions between these levels. These factors are arranged in the form of five nested layers with the most immediate layer having the most impact on their development. The family and the school environment fall within the most proximal environment for an adolescent, that is, the microsystem, and hence have the most influence on their behavior [17]. A study by [18] demonstrated that adolescents who have positive relationships with their parents display more social competence and fewer conduct disorders, while those with family disputes exhibit more tendency to antisocial conduct and maladaptive behaviors. These kinds of behavior can also be extended to Internet addiction. According to Jessor & Jessor’s [19] problem behavior theory, De Leo & Wulfert [20] demonstrated that problematic family relationships have a significant impact on the initiation and escalation of Internet addiction among adolescents. A study by [21] found that Chinese adolescents who reported a lack of parental affection and poor family environment were more likely to engage in problematic Internet use.

Literature shows a difference in early and middle adolescents’ cognitive and psychological development and how they interact with their families and peers [22]. Early adolescents appear to employ more emotion-focused coping techniques than older adolescents, who in turn employ more problem-focused coping techniques [23]. Therefore, when studying adolescent Internet addiction, it is crucial to examine the various developmental stages individually, considering the unique cognitive, psychological, and coping differences between early and middle adolescents, as they interact with their families and peers.

According to the stage-environment fit hypothesis in [24], adolescents perform better when the educational environment is tailored to their developmental requirements, and there are negative repercussions when it is not. In accordance with this hypothesis, several research studies demonstrated that the school environment predicted adolescent problems including Internet addiction [25, 26]. Chang and his colleagues discovered in a longitudinal research that children with negative school bonds were more likely to develop Internet addiction [27]. Furthermore, poor academic performance has been found to be associated with the risk of Internet addiction [28, 29]. Previous research found that adolescents with poor academic performance received less respect from their peers, and an adolescent’s poor academic performance may be connected with low self-esteem and behavioral issues such as sleep disorders, depression, low locus of control, suicidal thoughts, and antisocial personality disorder [30, 31]. These adolescents may turn to the Internet in pursuit of fulfillment and self-satisfaction as a result of their unhappiness with real-world circumstances and may be at a higher risk of Internet addiction.

Teachers are most likely to be a significant source of social support for adolescents in the school environment. Internet use was more prevalent among adolescents who received less social support from teachers. In addition, it has been shown that adolescents’ perceptions of their teachers’ support buffer the detrimental impacts of unpleasant or stressful environmental situations [32]. Students who believe their teachers are helpful, responsive, and concerned about them do better academically and have less problematic behaviors [33]. We hypothesize that positive teacher support may help with minimizing IA in students.

In the realm of psychology, the majority of research samples are drawn from Western, educated, industrialized, rich, and democratic populations, commonly referred to as WEIRD [34]. [35] observed that no Middle Eastern sample-based studies were published in a high-impact international psychology journal in 2014, and this tendency persisted in their 2017 follow-up analysis. This study is among the few to focus on a population from Middle Eastern countries, while most research has primarily focused on Western and East Asian populations [36, 37]. Given that cultural differences can affect research outcomes [38], this study on adolescent Internet addiction and its association with family and school environments contributes significantly to the field of psychology by offering original research using a non-WEIRD sample. Although different studies have examined the effects of family, school environment, and peer relationships on adolescent Internet addiction separately [39, 40], research examining them together is limited. Our study attempts to bridge this gap. By studying these models together, we aimed to explore the relative impact of family, school environment, and peer relationships on adolescent IA. Specifically, we aimed to identify the factors that had the most significant impact on the development of IA among adolescents. Understanding the combined effects of these microsystems is crucial for developing a comprehensive understanding of the factors contributing to IA in adolescents.

Numerous elements in the adolescent environment may function as factors affecting adolescents’ IA. Existing research regarding adolescent IA about family factors mainly takes into consideration parental monitoring, parental IA, and parenting styles [41, 42]. Considering the interplay between these factors across all layers of the socioecological model, including the multiple components of family, and school environment, will provide more thorough and ecologically sound findings. Examining the effects of a single factor or components from a single domain may easily be inflated since in practice factors from several environmental subsystems interact in synergistic ways [43].

A systematic review of longitudinal studies examining trends in IA found that most of the included studies examined individual-level factors in relation to IA. Among the examined 27 variables in the systematic review, only 4 variables constituted contextual factors (i.e. family, peers, and classroom-related factors) [44]. Given that individual factors have been extensively researched and to gain a deeper and more wholesome understanding of the IA phenomenon, this study aimed at exploring the relationship between age, family, and school-related factors and IA and its symptoms among a group of adolescents in Qatar. In our study, we used a survey to collect data. The survey included various measures to capture the relevant aspects of family functioning, school environment, and relationships with peers and teachers. To evaluate family functioning, we used the Brief Family Relationship Scale (BFRS) [45]. This scale measures perceived family cohesion, expressiveness, and conflict. Additionally, we incorporated selected questions from the WHO Health Behaviour in School-aged Children (HBSC) survey to provide valuable contextual information on various aspects of adolescent well-being and behaviors within the school context, including pressure at school, school workload, academic performance, relationships with peers, and teachers [46]. To assess Internet addiction symptoms, we utilized the Internet Addiction Diagnostic Questionnaire (IADQ) [3]. This study aims to answer the following research questions:

### RQ1

Is there an association between adolescents’ age, family functioning, parents’ employment status, school environment, relationship with teachers, relationship with peers, and adolescents’ IA?

### RQ2

Is there an association between adolescents’ age, family functioning, parents’ employment status, school environment, relationship with teachers, relationship with peers, and each symptom of IA?

## Methods

### Participants

Participants of this study were school students living in Qatar. The participants were recruited through online surveys carried out on SurveyMonkey. The link to the questionnaire was shared with adolescent students studying at 16 public and private schools in Qatar. The period of data collection was between March and May 2022. After excluding participants who did not meet the inclusion criteria of the study e.g. provide incomplete answers to the questions, 479 of the 586 students were included in the analytical sample of this study. 94 boys (19.62%) and 385 girls were in the sample (80.37%). The data collection was carried out during the period of final exams. Hence, some of the schools were more responsive than others. Among the schools that responded, some responses came in from two schools that had a large cohort of female students. The average age of adolescents was 13.21 (*SD* = 1.23, range: 11 to 17). At the time of data collection, participants were informed of the study’s aim, that participation is voluntary, and that they are free to refrain from answering any of the questions or withdraw from the survey at any point. Additionally, the survey was distributed in both English and Arabic. To ensure the quality of translation and the original meaning is preserved, the Arabic survey was developed using the back-translation method. The study was approved by the first author’s Institutional Review Board and schools permissions were obtained to distribute the survey.

### Measures

The survey consisted of two parts. The first part included questions on the child’s demographics. The second part included questions on the adolescents’ level of Internet Addiction, family environment, school environment, relationship with teachers and peers, and academic performance.

#### **Employment Status of both parents**

Adolescents were asked to answer questions on the employment status of their mother and father. To compute the binary variable Both Parents Employed, the responses to each parent’s employment status were combined and coded as “Yes” if both parents were employed and “No” if only one of the parents was employed.

#### **Age**

Adolescents were asked to explicitly report their age. Based on their responses, they were classified into Early adolescents if they were in the age group 11–13, and Middle Adolescents if they were in the age group 14–17. The classification of adolescents into the developmental phases of early and middle adolescents was made based on literature [47, 48]. Only five students in our sample had reported that they were 17 years of age, and may have reported so on completing 16 years of age.

#### **Internet Addiction Diagnostic Questionnaire (IADQ)**

IADQ is an eight-item questionnaire (binary response format: no, yes), each of the questions representing a symptom for detecting Internet addiction [3]. The IADQ total score (ranging from 0 to 8) is calculated by adding the values of each of the eight binary questions. According to Young, participants who answered “yes” to five or more of the symptoms, were classified as addicted Internet users, while the others as non-addicted Internet users. The questions in IADQ assess the existence of the following criteria (symptoms) of Internet Addiction: “preoccupation” (Q1), “tolerance” (Q2), “unsuccessful efforts to limit or stop Internet usage” (Q3), “withdrawal” (Q4), “loss of control of time spent on the Internet” (Q5), “risk/ lose relationships/opportunities” (Q6), “lies to conceal the extent of involvement” (Q7), and “dysfunctional coping” (Q8) [49]. Prior studies on the IADQ reported Cronbach’s alpha values ranging from 0.60 to 0.72 [50]. The reliability of the IADQ in this study was deemed acceptable, with a Cronbach’s alpha value of 0.62 [51].

#### **Brief family relation scale (BFRS)**

Family environment was measured using the total score of the Brief Family Relation Scale (BFRS) [45]. This scale has been adopted from the 27-item Relationship dimension of the Family Environment Scale (FES) [52] and consists of 16 items. Each item on the BFRS scale is answered using a three-point scale (1 = ‘Not at all’, 2 = ‘Sometimes’, and 3 = ‘A lot’). The total BFRS score (ranging from 16 to 48) is calculated by adding the values of each of the responses. A higher score indicates a better family relationship. The BFRS provides perceptions of family functioning and consists of three subscales – family cohesion, family conflict-, and expressiveness. The reliability of BFRS in this study was deemed very good, with a Cronbach’s alpha value of 0.88 [51].

#### **School environment, academic performance, teacher support, and peer relationship**

were measured using the questions on the school setting from “ Health Behaviour in School-aged Children: WHO Collaborative Cross-National survey/study (HBSC) 2013–2014” (HBSC) [46]. The Health Behaviour in School-Aged Children (HBSC) project is a collaborative, cross-national study performed by the World Health Organization (WHO) among adolescents aged between 11 and 15 from over 40 nations and regions in Europe and North America. Table 1 provides a brief of each of the variables that were included from the HBSC. In addition to the questions from HBSC, an additional question on education performance was also included.

**Table 1:** Overview of Used Variables with regard to the School Environment, Academic Performance, Teacher Relationship, and Peer Relationship

Variable (group)	Question	Response categories along with their numerical coding	Reference
Education Performance (Academic Performance)	How is your education performance in school?	[4] Very Good [3] Good [2] Average [1] Below Average	
School feeling (School Environment)	How do you feel about school at present?	[4] I like it a lot [3] I like it a bit [2] I don’t like it very much [1] I don’t like it at all	These questions were adapted from the section on school settings in HBSC, [46] )
School pressure (School Environment)	How pressured do you feel by the schoolwork/homework you have to do?	[4] Not at all [3] A little [2] Some [1] A lot	
Schoolwork problem (School Environment)	In general, how much of a problem have you had getting your schoolwork/homework done on time?	[4] No problem [3] Some problem [2] A considerable problem [1] A serious problem	
School comparative performance (Academic performance)	In your opinion, what does your class teacher(s) think about your school performance compared to your classmates?	[4] Very good [3] Good [2] Average [1] Below average	
Class enjoyment (peer support)	The students in my class(es) enjoy being together	[5] Strongly agree [4] Agree [3] Neither agree or disagree [2] Disagree [1] Strongly disagree	
Class kind help (peer support)	Most of the students in my class(es) are kind and helpful		
Class acceptance (peer support)	Other students accept me as I am		
Teacher acceptance (teacher support)	I feel that my teachers accept me as I am		
Teacher caring (teacher support)	I feel that my teachers care about me as a person		
Teacher trust (teacher support)	I feel a lot of trust in my teachers		

### Statistical analysis

Exploratory factor analysis with orthogonal (Varimax) rotation was used to identify the latent structure of the school environment variables in our study [53]. Using the following criterion, the number of significant components to be preserved for rotation was established: (1) The scree plot had to be consistent with the amount of extracted factors, and (2) the factor solution had to allow for a coherent interpretation.

Multiple linear regression analysis was conducted to identify the relationship between family relationships, school environment, academic performance, peer support, teacher support, employment status of parents, and age with adolescents’ IA. Internet addiction status was considered as the outcome variable.

Binary logistic regression was also conducted to identify the relationship between family environment, school environment, academic performance, peer support, teacher support, employment status of parents, and age with the presence of each of the Internet addiction symptoms in adolescents. The significance level was set at 0.05. All statistical analyses were performed using JASP version 0.16.3 [54].

## Results

### Participant demographics

Descriptive statistics of the participants are shown in Table 2. The prevalence of each of the Internet addiction symptoms in adolescents is described in Table 3.

**Table 2:** Descriptive Statistics of Demographic Variables and Internet addiction, BFRS, and HBSC variables

*Variable*		*n*		*%*
**Are both parents employed?**				
Yes		260		54.28
No		219		45.72
**Age**				
Early Adolescence		298		62.21
Middle Adolescence		181		37.87
**Internet Addiction Prevalence in Adolescents**				
Addicted Internet Users		142		29.64
Non – Addicted Internet Users		337		70.36
**Family, School, IA variables**		***M***		***SD***
Total Family relationship (BFRS)		38.82		6.28
Total IA		3.38		1.98
Education Performance		3.42		0.71
School feeling		3.02		0.83
School pressure		2.74		0.94
Schoolwork problem		2.06		0.85
School comparative performance		3.31		0.73
Class enjoyment		4.19		0.83
Class kind help		3.89		1.02
Class acceptance		4.07		1.02
Teacher acceptance		4.06		1.04
Teacher caring		3.89		1.00
Teacher trust		3.85		1.17

**Table 3:** Prevalence of IA Symptoms in Adolescents

Internet addiction symptoms	*n* (%)	
	Yes	No
Preoccupation	238 (49.69%)	241 (50.31%)
Tolerance	150 (31.32%)	329 (68.69%)
Made unsuccessful efforts to control Internet use repeatedly	212 (44.26%)	267 (55.74%)
Withdrawal	189 (39.46%)	290 (60.54%)
Staying online longer than intended	294 (61.38%)	185 (38.62%)
Risk/ lose relationships/opportunities because of the Internet	113 (23.59%)	366 (76.41%)
Lies to conceal extent of involvement	111 (23.17%)	368 (76.83%)
Dysfunctional coping	311 (64.93%)	168 (35.07%)

### Factor analysis of school environment variables

To group the items of the school environment variables into factor scores, the data set of the sample was subjected to an exploratory factor analysis. The Kaiser-Meyer-Olkin (KMO), a Measure of Sampling Adequacy (MSA), was employed in this research to evaluate multicollinearity in the data so that the feasibility of conducting a factor analysis could be determined. The overall KMO measure was 0.82 with individual KMO measures all greater than 0.7, classifications of ‘middling’ to ‘marvellous’ according to Kaiser [55]. Bartlett’s test of sphericity was statistically significant (p < .0001), indicating that the data was likely factorizable.

The analysis extracted four components which accounted for 53% of the variance. Our scree plot was also found compatible with a four-factor solution. We interpreted the four factors as they are representing the following variables of school environment measurement. Factor 1 (16% of total variance): teacher support, factor 2 (14%): peer support, factor 3 (12%): academic performance, and factor 4 (11%): school environment. Factor loadings for factor 1, factor 2, factor 3, and factor 4 ranged from 0.567 to 0.767, 0.635 to 0.684, 0.536 to 0.924, and 0.445 to 0.635 respectively. The factor scores were derived by adding the scores of individual items within each empirical domain (sum score) and dividing these sum scores by the total number of items (mean item score). Component loadings and uniqueness of the rotated solution are presented in Table 4. The reliability of the school environment, teacher support, peer support and academic performance in this study was deemed acceptable, with a Cronbach’s alpha value of 0.68, 0.78, 0.73, and 0.73 respectively.

**Table 4:** Factor Analysis of School Environment Variables

Items	Factor 1	Factor 2	Factor 3	Factor 4	Uniqueness
Teacher caring	0.767				0.31
Teacher trust	0.693				0.39
Teacher acceptance	0.567				0.61
Class acceptance		0.684			0.48
Class kind help		0.681			0.52
Class enjoyment		0.635			0.53
School comparative performance			0.924		0.01
Education performance			0.536		0.63
School pressure				0.635	0.52
School work problem				0.609	0.54
School feeling				0.445	0.63

### (RQ1) predictors of adolescents’ IA

Multiple regression analysis was used to determine the factors in the current study that predict adolescents’ total IA scores. No outliers were found in the data. Pearson’s correlation was also used to analyze associations between the total IA which was the dependent variable, and the independent variables of Total BFRS score, teacher support, peer support, academic performance, school environment, age, and parents’ employment statuses. All assumptions of linearity, normality, homoscedasticity, and multicollinearity were satisfied. The correlations between the continuous variables is shown in Table 5. We note here the relatively higher correlation between teacher support and school environment.

**Table 5:** Correlation between Adolescent Total IA and other Independent Variables

Pearson’s correlation						
Variable	1	2	3	4	5	6
1. Total IA	—					
2. Total BFRS score	-0.38***	—				
3. School environment	-0.31***	0.44***	—			
4. Academic Performance	-0.20***	0.25***	0.41***	—		
5. Peer support	-0.15**	0.30***	0.35***	0.25***	—	
6. Teacher support	-0.11**	0.30***	0.51***	0.43***	0.33***	

* p < .05, ** p < .01, *** p < .001

Table 6 demonstrates the results of the multiple regression that was run to predict the total Internet Addiction score from the total BFRS score, academic performance, peer support, teacher support, both parents’ employment status, and school environment. There was independence of residuals, as assessed by a Durbin-Watson statistic of 1.81. There was no evidence of multicollinearity, as assessed by tolerance values greater than 0.1. The assumption of normality was met, as assessed by a Q-Q plot. The multiple regression model statistically significantly predicted the total IA score, *F (7, 471) = 15.53, p < .001*, R^2^ = 0.19, adjusted R^2^ = 0.18. Within the model, total BFRS score (*β = -0.31, p < .001*), teacher support (*β = 0.12, p = .017)*, and school environment (*β = -0.20, p < .001)* were the only significant predictors of the adolescents’ total IA score.

**Table 6:** Multiple Linear Regression Analysis predicting Total IA score

	*R* ^*2*^	*Adjusted R* ^*2*^	*F (df)*	
	0.188	0.175	15.534 (7, 471)	
**Predictors**	***Standardized β***	***t***	***p***	
Constant		37.35	< 0.001	
Age (Early: 1, Middle: 2 )	0.06	1.31	0.189	
Both Parents’ employment status (No: 0, Yes: 1)	-0.05	-1.20	0.268	
Total BFRS	-0.31	-6.56	< 0.001	
Academic Performance	-0.08	-2.17	0.078	
Peer support	-9 × 10^− 3^	-0.19	0.844	
Teacher support	0.12	2.39	0.017	
School environment	-0.20	-3.67	< 0.001	

### (RQ2) predictors of the individual symptoms of adolescent IA

#### IA symptom of preoccupation

Binomial logistic regression was performed to ascertain the effects of total BFRS score, academic performance, peer support, teacher support, both parents’ employment status, and school environment on the likelihood that adolescents exhibited the Internet addiction symptom of preoccupation. The logistic regression model was statistically significant, χ2(471, *N* = 479) = 22.05, p = .002. The model explained 6.0% (Nagelkerke R^2^) of the variance in the IA symptom of preoccupation and correctly classified 59.92% of cases. Sensitivity was 57%, and specificity was 62.7%. Of the seven predictor variables, only three were statistically significant: total BFRS, academic performance, and teacher support (as shown in Table 7). Increasing BFRS score, and increasing academic performance were associated with a reduction in the likelihood of exhibiting the IA symptom of preoccupation (OR = 0.95, 95% CI [0.92, 0.99]), (OR = 0.70, 95% CI [0.50, 0.97]), respectively, but increasing teacher support was associated with an increased likelihood of exhibiting the IA symptom of preoccupation (OR = 1.43, 95%CI [1.11, 1.86]).

**Table 7:** The Predictive Factors for Adolescent IA Symptom of Preoccupation

Predictors	SE	B	OR	Wald	df	95% CI for Odds Ratio		p
						Lower	Upper	
(Intercept)	0.82	-0.01	5.39	4.23	1	1.08	26.81	0.040
Age (Early: 1, Middle: 2 )	0.20	-0.00	0.99	1 × 10^− 3^	1	0.68	1.46	0.974
Both Parents’ employment status (No: 0, Yes: 1)	0.19	0.06	1.13	0.43	1	0.78	1.64	0.513
Total BFRS	0.02	-0.31	0.95	7.64	1	0.92	0.99	6 × 10^− 3^
School environment	0.18	-0.15	0.80	1.54	1	0.56	1.14	0.215
Academic Performance	0.17	-0.23	0.70	4.46	1	0.50	0.97	0.035
Peer support	0.14	0.10	1.14	0.97	1	0.88	1.49	0.324
Teacher support	0.13	0.32	1.43	7.35	1	1.11	1.86	0.007

#### IA symptom of tolerance

The logistic regression model was statistically significant, χ2(471, *N* = 479) = 19.733, p = .006. The model explained 5.7% (Nagelkerke R^2^) of the variance in the IA symptom of tolerance and correctly classified 70.15% of cases. Sensitivity was 10%, and specificity was 97.6%. Of the seven predictor variables, only one was statistically significant: total BFRS (as shown in Table 8). Increasing BFRS score was associated with a reduction in the likelihood of exhibiting the IA symptom of tolerance (OR = 0.97, 95% CI [0.93, 1.00]).

**Table 8:** The Predictive Factors for Adolescent IA symptom of Tolerance

Predictors	SE	B	OR	Wald	df	95% CI for Odds Ratio		*p*
						Lower	Upper	
(Intercept)	0.85	-0.82	4.61	3.23	1	0.87	24.41	0.072
Age (Early: 1, Middle: 2 )	0.21	0.08	1.18	0.64	1	0.78	1.79	0.423
Both Parents’ employment status (No: 0, Yes: 1)	0.21	0.11	1.25	1.15	1	0.83	1.87	0.284
Total BFRS	0.02	-0.22	0.97	3.89	1	0.93	1.00	0.049
School environment	0.19	-0.15	0.80	1.38	1	0.55	1.16	0.239
Academic Performance	0.18	-0.08	0.88	0.55	1	0.62	1.24	0.457
Peer support	0.14	-0.20	0.78	3.23	1	0.59	1.02	0.072
Teacher support	0.14	0.17	1.21	1.85	1	0.92	1.59	0.174

#### IA symptom of unsuccessful efforts to control internet use

The logistic regression model was statistically significant, χ2(471, *N* = 479) = 21.67, p = .003. The model explained 5.9% (Nagelkerke R^2^) of the variance in the IA symptom of unsuccessful efforts to control Internet use and correctly classified 56.99% of cases. Sensitivity was 31.1%, and specificity was 77.5%. Of the seven predictor variables, only one was statistically significant: total BFRS (as shown in Table 9). Increasing BFRS score was associated with a reduction in the likelihood of exhibiting the IA symptom of unsuccessful efforts to control Internet use (OR = 0.94, 95% CI [0.91, 0.98]).

**Table 9:** The Predictive Factors for Adolescent IA Symptom of Unsuccessful Efforts to Control Internet Use in Adolescents

Predictors	SE	B	OR	Wald	df	95% CI for Odds Ratio		p
						Lower	Upper	
(Intercept)	0.83	-0.24	13.19	9.78	1	2.62	66.43	0.002
Age (Early: 1, Middle: 2)	0.20	0.01	1.02	0.01	1	0.69	1.50	0.934
Both Parents’ employment status (No: 0, Yes: 1)	0.19	-0.11	0.80	1.43	1	0.55	1.16	0.232
Total BFRS	0.02	-0.36	0.94	10.82	1	0.91	0.98	0.001
School environment	0.18	-0.07	0.91	0.29	1	0.64	1.29	0.589
Academic Performance	0.17	-0.19	0.75	3.02	1	0.54	1.04	0.083
Peer support	0.14	0.02	1.02	0.03	1	0.78	1.33	0.875
Teacher support	0.13	0.16	1.19	1.75	1	0.92	1.54	0.186

#### IA symptom of withdrawal

The logistic regression model was statistically significant, χ2(471, *N* = 479) = 51.49, p < .001. The model explained 13.8% (Nagelkerke R^2^) of the variance in the IA symptom of withdrawal and correctly classified 66.81% of cases. Sensitivity was 38.1%, and specificity was 85.5%. Of the seven predictor variables, only three were statistically significant: total BFRS, school environment, and teacher support (as shown in Table 10). Increasing BFRS score, and increasing school environment was associated with a reduction in the likelihood of exhibiting the IA symptom of withdrawal (OR = 0.92, 95% CI [0.89, 0.96]), (OR = 0.55, 95% CI [0.38, 0.80]), but increasing teacher support was associated with an increased likelihood of exhibiting the IA symptom of withdrawal in adolescents (OR = 1.41, 95% CI [1.07, 1.87]).

**Table 10:** The Predictive Factors for Adolescent IA Symptom of Withdrawal

Predictors	SE	B	OR	Wald	df	95% CI for Odds Ratio		p
						Lower	Upper	
(Intercept)	0.87	-0.47	28.88	15.25	1	5.34	156.20	9.45 × 10^− 5^
Age (Early: 1, Middle: 2 )	0.21	-0.01	0.98	0.01	1	0.66	1.48	0.935
Both Parents’ employment status (No: 0, Yes: 1)	0.20	-0.18	0.69	3.29	1	0.47	1.03	0.070
Total BFRS	0.02	-0.52	0.92	20.02	1	0.89	0.96	7.66 × 10^− 6^
School environment	0.19	-0.41	0.55	9.79	1	0.38	0.80	0.002
Academic Performance	0.18	-0.10	0.86	0.78	1	0.61	1.21	0.376
Peer support	0.14	0.08	1.10	0.46	1	0.83	1.45	0.496
Teacher support	0.14	0.31	1.41	5.90	1	1.07	1.87	0.015

#### IA symptom of staying online longer than intended

The logistic regression model was statistically significant, χ2(471, *N* = 479) = 37.70, p < .001. The model explained 10.3% (Nagelkerke R^2^) of the variance in the IA symptom of staying online longer than intended and correctly classified 65.55% of cases. Sensitivity was 84.7%, and specificity was 35.1%. Of the seven predictor variables, only three were statistically significant: total BFRS, both parents’ employment status, and school environment (as shown in Table 11). Increasing BFRS score, and increasing school environment score was associated with a reduction in the likelihood of exhibiting the IA symptom of staying online longer than intended (OR = 0.94, 95% CI [0.91, 0.98]), (OR = 0.64, 95% CI [0.44, 0.93]). Adolescents with only one employed parent had higher odds of staying online longer compared to those with parents who are both employed (OR = 0.62, 95% CI [0.42, 0.91]).

**Table 11:** The Predictive Factors for Adolescent IA Symptom of Staying Online Longer than Intended

Predictors	SE	B	OR	Wald	df	95% CI for Odds Ratio		p
						Lower	Upper	
(Intercept)	0.91	0.51	56.95	19.64	1	9.53	340.33	9.37 × 10^− 6^
Age (Early: 1, Middle: 2 )	0.21	0.08	1.18	0.66	1	0.79	1.77	0.416
Both Parents’ employment status (No: 0, Yes: 1)	0.20	-0.24	0.62	5.83	1	0.42	0.91	0.016
Total BFRS	0.02	-0.36	0.94	9.12	1	0.91	0.98	0.003
School environment	0.20	-0.31	0.64	5.56	1	0.44	0.93	0.018
Academic Performance	0.18	0.03	1.05	0.08	1	0.74	1.49	0.779
Peer support	0.14	0.04	1.06	0.16	1	0.80	1.40	0.691
Teacher support	0.14	-0.10	0.89	0.67	1	0.68	1.17	0.415

#### IA symptom of risking/losing opportunities because of the internet

The logistic regression model was statistically significant, χ2(471, *N* = 479) = 17.34, p = .015. The model explained 5.3% (Nagelkerke R^2^) of the variance in the IA symptom of risking/losing opportunities because of the Internet and correctly classified 77.04% of cases. Sensitivity was 3%, and specificity was 100%. Of the seven predictor variables, only one was statistically significant: total BFRS (as shown in Table 12). Increasing BFRS was associated with a reduction in the likelihood of exhibiting the IA symptom of risking/losing opportunities because of the Internet (OR = 0.95, 95% CI [0.92, 0.99]).

**Table 12:** The Predictive Factors for Adolescent IA Symptom of Risking/Losing Opportunities Because of the Internet

Predictors	SE	B	OR	Wald	df	95% CI for Odds Ratio		p
						Lower	Upper	
(Intercept)	0.91	-1.23	3.71	2.09	1	0.63	21.96	0.149
Age (Early: 1, Middle: 2)	0.23	0.11	1.25	0.93	1	0.80	1.95	0.335
Both Parents’ employment status (No: 0, Yes: 1)	0.22	-0.01	0.99	0.00	1	0.64	1.53	0.954
Total BFRS	0.02	-0.31	0.95	6.82	1	0.92	0.99	0.009
School environment	0.21	-0.11	0.85	0.62	1	0.57	1.27	0.430
Academic Performance	0.19	-0.21	0.72	2.92	1	0.50	1.05	0.087
Peer support	0.16	0.05	1.06	0.15	1	0.78	1.44	0.699
Teacher support	0.15	0.09	1.10	0.42	1	0.82	1.48	0.516

#### IA symptom of lying to conceal the extent of involvement

The logistic regression model was statistically significant, χ2(471, *N* = 479) = 43.97, p < .001. The model explained 13.0% (Nagelkerke R^2^) of the variance in the IA symptom of lying to conceal the extent of involvement and correctly classified 76.83% of cases. Sensitivity was 9%, and specificity was 97.28%. Of the seven predictor variables, four were statistically significant: age, total BFRS, school environment, and teacher support (as shown in Table 13). Increasing BFRS score, and increasing school environment was associated with a reduction in the likelihood of exhibiting the IA symptom of lying to conceal extent of involvement (OR = 0.93, 95% CI [0.90, 0.97]), (OR = 0.56, 95% CI [0.37, 0.86]), but increasing teacher support was associated with an increased likelihood of exhibiting the IA symptom of lying to conceal the extent of involvement (OR = 1.49, 95% CI [1.09, 2.04]). Middle adolescents had higher odds of exhibiting the symptom of lying to conceal extent of involvement as compared to early adolescents (OR = 1.73, 95% CI [1.09, 2.73]).

**Table 13:** The Predictive Factors for Adolescent IA Symptom of Lying to Conceal the Extent of Involvement

Predictors	SE	B	OR	Wald	df	95% CI for Odds Ratio		p
						Lower	Upper	
(Intercept)	0.93	-1.33	7.03	4.42	1	1.14	43.30	0.036
Age (Early: 1, Middle: 2 )	0.23	0.27	1.73	5.43	1	1.09	2.73	0.020
Both Parents’ employment status (No: 0, Yes: 1)	0.23	-0.06	0.89	0.25	1	0.57	1.40	0.617
Total BFRS	0.02	-0.44	0.93	12.70	1	0.90	0.97	3.67 × 10^− 4^
School environment	0.22	-0.39	0.56	7.22	1	0.37	0.86	0.007
Academic Performance	0.20	-0.19	0.75	2.24	1	0.51	1.10	0.135
Peer support	0.16	-0.05	0.94	0.15	1	0.69	1.28	0.696
Teacher support	0.16	0.35	1.49	6.10	1	1.09	2.04	0.014

#### IA symptom of dysfunctional coping

The logistic regression model was statistically significant, χ2(471, *N* = 479) = 49.65, p < .001. The model explained 13.6% (Nagelkerke R^2^) of the variance in the IA symptom of dysfunctional coping and correctly classified 68.27% of cases. Sensitivity was 88.42%, and specificity was 30.95%. Of the seven predictor variables, only two were statistically significant: total BFRS, and school environment (as shown in Table 14). Increasing BFRS scores, and increasing school environment scores were associated with a reduction in the likelihood of exhibiting the IA symptom of dysfunctional coping (OR = 0.93, 95% CI [0.90, 0.97]), (OR = 0.58, 95% CI [0.40, 0.86]).

**Table 14:** The Predictive Factors for Adolescent IA Symptom of Dysfunctional Coping

Predictors	SE	B	OR	Wald	df	95% CI for Odds Ratio		p
						Lower	Upper	
(Intercept)	0.98	0.71	163.15	26.86	1	23.76	1120.16	2.18 × 10^− 7^
Age (Early: 1, Middle: 2 )	0.21	0.06	1.14	0.37	1	0.75	1.73	0.542
Both Parents’ employment status (No: 0, Yes: 1)	0.20	-0.00	1.00	8.6 × 10^− 7^	1	0.67	1.49	0.999
Total BFRS	0.02	-0.45	0.93	12.08	1	0.90	0.97	5.10 × 10^− 4^
School environment	0.20	-0.37	0.58	7.46	1	0.40	0.86	0.006
Academic Performance	0.19	0.17	1.31	2.16	1	0.92	1.89	0.142
Peer support	0.15	-0.15	0.83	1.59	1	0.61	1.11	0.207
Teacher support	0.14	-0.11	0.89	0.71	1	0.67	1.18	0.400

## Discussion

Guided by Bronfenbrenner’s socioecological model, our study examined factors within the microsystem of adolescents related to an important health issue that has been long affecting adolescents. Internet addiction is a serious behavioral addiction warranting a deeper understanding that extends beyond individual factors. In this study, we examined if family-related factors and school-related factors are associated with Internet addiction. We further explored if these microsystem-related factors are associated with each Internet addiction symptom. The findings from the multiple regression model suggest that family relationship, school environment, and teacher support are significant predictors of Internet addiction in adolescents.

Our results demonstrate that family relationships can affect Internet addiction. The perception of poor family relationships was found to be a significant predictor of all IA symptoms. This relationship can be further understood through the lens of the escape from self-theory. Escape from self-theory suggests that individuals may turn to excessive Internet use as a means of escaping or avoiding problems in their real-life relationships, particularly within the family. This implies that individuals who perceive their family relationships negatively are more likely to exhibit problematic Internet use patterns, such as excessive time spent online, difficulty controlling their online activities, neglecting responsibilities, and experiencing negative consequences. Literature has long established that family characteristics can influence addictive behaviors in adolescents [56, 57]. Tafa & Baiocco found that adolescents with families who are rigid and lack cohesion in their response to stressful situations have higher addiction rates [57]. Poor family functioning such as high levels of conflict or poor cohesiveness may lead adolescents to excessive use of the Internet as means of escaping or a distracting mechanism. Understanding the connection between poor family relationships and IA symptoms sheds light on the underlying psychological mechanisms. It emphasizes the importance of addressing family dynamics and improving family relationships as potential avenues for intervention and prevention strategies in combating Internet addiction.

Our findings are in line with that of Kim et al. who reported that family dysfunction, specifically among adolescents who’ve experienced domestic violence, was a significant predictor of smartphone addiction [58]. Our findings are also in line with several other studies that examined family function in relation to Internet addiction [59, 60]. A study by Şenormancı et al. examined 30 male adult patients admitted to an Internet addiction clinic [59] and found that when compared to the control group, patients with IA addiction evaluated their family functioning as poor and problematic. Similarly, another study found that college students with severe IA reported perceptions of low family functioning compared to non-addicted students [60]. The fact that these effects can be seen in adolescents is alarming and may indicate the long-lasting effects of problematic family functioning on addictive behaviors.

These results may also be further explained according to the escape from self-theory [61], when individuals perceive a discrepancy between their current situations and expectations, they seek to escape from the self (i.e., self-awareness) to eliminate negative reactions and emotions by engaging in self-destructive behaviors. The Internet may serve as an escapism mechanism for adolescents with such perceptions as seen in other studies. Findings from Kwon et al.’s research suggest that adolescents addicted to Internet gaming have become so as an attempt to escape from their own self and reality [62].

In our study, we found that adolescents with only one employed parent had higher odds of staying online longer compared to adolescents with both parents working. It might be that in families with one parent employed, the parents staying at home might be spending longer time online. In a previous study, parents’ Internet behaviors online reflected on their children [42]. The study found that adolescents had higher chances of being dependent Internet users when their parents were dependent Internet users. The authors also found that the same displayed IA symptoms in parents were reflected in their adolescents’ Internet-related behaviors.

Our study indicates that adolescents with only one employed parent tend to spend more time online, and it is possible that the parent staying at home contributes to this behavior. Furthermore, there are studies examining the role of social support as an important protective factor in adolescents. In a survey study examining IA and perceived social support conducted among adolescents attending a clinic for adolescents’ behavioral issues, adolescents with a working mother had a significantly higher perception of social support compared to adolescents whose mothers did not work. Additionally, adolescents who reported spending time with their mothers by engaging in various activities also reported higher social support and a significantly lower IA [63]. Taking into consideration the collectivistic nature of the local culture in which the father is the main provider for the family, we assume that the father is the working parent among adolescents reporting only one parent working. It is important to consider family dynamics and parental behavior when addressing IA and promoting healthy Internet use among adolescents. The study highlights the need for a holistic approach to addressing IA in adolescents by taking into account the complex interplay between family dynamics, parental employment status, and Internet use.

The school environment is an essential part of the microsystem of Bronfenbrenner’s ecological framework. Adolescents’ perceptions of their school might be shaped by stressful or unpleasant experiences such as conflicts and rejections or positive self-enhancing experiences such as teacher’s support and other successes that adolescents go through and experience [64]. These experiences in school that shape adolescents’ perceptions can have a detrimental effect on their psychosocial development. There’s a plethora of evidence suggesting the impact of adolescents’ perceptions of school climate on psychological adaptive behaviors and student development. A study by Wang & Dishion that followed 1030 students from sixth to eighth grade [32] and measured their perceptions towards school climate found a decline in students’ perceptions of their school climate over time across all dimensions, while they observed an increase in behavioral problems and misbehaving peers in return [32]. Another longitudinal study that examined 1451 students from sixth through eighth grade also found that students perceived school climate declined over that period. This decline was associated with a decline in psychological and social adjustment among adolescents [65].

When students’ perceptions of their school climate decrease, they are more likely to lose the sense of belonging, and the sense of community, creating distress and thus making them more vulnerable to problematic behaviors as observed in our study. The school environment in our study was measured by adolescents’ perceptions and feelings towards their teachers and school-related work stress. Adolescents in our study with negative attitudes towards the school environment predicted higher rates of IA in addition to higher reported symptoms of withdrawal, staying online longer than intended, lying to conceal the extent of IA, and dysfunctional coping. For these adolescents, the Internet might be serving as a dysfunctional coping mechanism that provides them with an escape from their reality. These findings are not surprising as they are in line with other studies. In a study examining high school students’ attitudes toward learning, the authors found that negative attitudes toward learning were positively associated with IA. The authors further explain that the inefficient use of technology could be an important factor in that association [66]. Another study of 2758 Chinese adolescents also found that negative school climate perceptions were positively associated with Internet addiction [25]. Multiple studies, however, suggest that for a more in-depth understanding of IA and as a future direction, the role of mediating factors should be further considered [25, 26].

The results of our study indicate that academic performance was associated with the IA symptom of withdrawal. A vast body of literature exists highlighting the negative impact IA has on academic performance. Not many studies however examined academic performance as a predictor of IA symptoms. A study that examined predictors of Internet addiction among a large group of 1914 adolescents in India, found that there was a weak negative correlation between academic performance and IA [67]. Furthermore, the authors found that individuals with IA had a lower academic performance compared to the group without IA addiction. Another study carried out in Turkey among 370 adolescents in 9th through 11th grade, found a significant difference in IA total scores between the students who perceived themselves as academically poor performers and those who perceived themselves as good performers in school in favor of those who reported poor school success [66]. Our findings are explainable since adolescents who perceive themselves as not performing well in school may use the Internet as a way to cope with stress and emotional difficulties, leading to excessive and unhealthy use. Conversely, students who perceive themselves as having high academic achievement may also have high self-efficacy and self-discipline that result in promoting positive habits and resilience, reducing the risk of developing Internet addiction. A study conducted among 489 Turkish adolescents found that the type of compulsive Internet use (task-related vs. non-task related) has various effects on adolescents’ perceived academic performance. The authors found that non-task-related compulsive Internet use is negatively associated with how adolescents perceive themselves academically. The study also found that task-related compulsive Internet use is positively predicted by adolescents’ perceived academic performance. This association was moderated by academic motivation [68]. Another study that examined social media use among 195 university students found that students’ academic performance predicted their social media usage [69]. Specifically, students with a lower GPA had a higher engagement with social media.

An intriguing finding that arose in our study is that teacher support was negatively correlated with IA. However, in the regression analysis, teacher support became a positive predictor of IA. We cautiously interpret this finding as teachers’ support may be acting as a proxy for students who may be struggling academically or struggling with IA as these students may truly view their teachers as lending extra support to them since they tend to need it the most. To further enrich our understanding, future research should investigate in greater depth the relationship between teacher support and IA. This approach would provide valuable insights into both students’ and teachers’ subjective experiences and perspectives, offering a more nuanced understanding of how support in schools can potentially exacerbate addictive behavior if provided excessively. Further research could investigate the potential unintended consequences of teacher support on IA, and how certain interventions may need to be tailored to individual student needs. Researchers may also consider examining IA in conjunction with other behaviors, such as academic and personal difficulties, and may view IA as a potential outcome rather than a primary instigator. It is essential to investigate how teachers’ support, which is frequently directed at struggling students, may inadvertently encourage them to view the Internet as a means of escape.

Our findings also validate the importance to investigate differences in Internet addiction in adolescence over the different age groups. Middle adolescents were more likely to exhibit the symptom of lying to conceal the extent of involvement as compared to early adolescents. This implies that changes in development and differences between different age groups can affect how parents, teachers, and health professionals help adolescents deal with IA. We hypothesize that parents of middle adolescents may have more relaxed or less parental controls such as limiting the time their children spend online compared to younger adolescents. A study found that among adolescents who exhibited IA symptoms, the majority of the parents in that adolescent group did not limit Internet use [37].

Due to the cross-sectional nature of the study, we cannot establish causal relationships between our predictor variables and IA. In addition, our sample consisted of early and middle adolescents and did not take into consideration, late adolescents. Another limitation was that answers were self-reported by adolescents, which might lead to reporting bias. The adolescents may not have accurately reported their academic performance and Internet addiction symptoms. Despite research indicating that there may be gender differences in adolescent Internet addiction [28, 70], we were unable to examine the effect of gender in our study as more than 80% of our sample were females. While our work was guided by Bronfenbrenner’s socioecological framework, we were only able to examine proximal factors as predictors of IA. To gain a better and more in-depth understanding, future studies can delve into the mesosystem level of Bronfenbrenner’s ecological model, which examines the interactions and linkages between these microsystems. For instance, the influence of the home environment on a child’s academic performance within the school setting can be further explored to understand how these microsystems mutually influence each other.

Despite the limitations, our study is the first of its kind in Qatar and the region to examine IA concerning adolescents’ age, family functioning, parents’ employment status, school environment, relationship with teachers, and relationship with peers. The findings shed light on an important growing health issue affecting adolescents that deserves serious attention from parents, schools, and policymakers. A notable strength of this study is its focus on a population from Middle Eastern countries, which is a significant contribution to the field since most prior research has primarily concentrated on Western and South Asian populations.

Academic performance can serve as an indicator of a student’s well-being and may allow for early identification of potential issues, such as IA. This information can then be used by policymakers and school administrators to implement effective interventions and preventive strategies for IA. Schools can play a leading role in preventing IA by spreading awareness of the dangers related to excessive Internet use, providing students with the tools needed to cultivate healthy habits related to Internet use, and fostering a school environment and culture built on a strong foundation of digital literacy from a very young age [71]. Digital literacy can be incorporated into school curricula. In fact, schools can also play an instrumental role in augmenting the digital literacy of families, especially parents, as parental awareness should be raised about the influence of their behavior in the context of healthy Internet use, along with the adoption of good digital literacy practices [72]. Family support, on the other hand, may offer adolescents a secure and caring setting in which to restrict their Internet usage and cultivate good connections and behaviors.

In terms of addressing existing IA symptoms, schools can be mandated to offer resources and services to support families struggling with adolescent IA. For example, such services could include counseling for students who may be struggling with these symptoms. A referral system can also be integrated between schools and healthcare professionals specializing in behavioral addictions to offer the support needed to adolescents exhibiting IA symptoms and their families. Encouraging teachers to play a proactive role in recognizing the signs of IA is also important. This could be achieved by training teachers and even parents to recognize the early signs of IA and how to intervene properly [71]. School policies can also be established to restrict students’ access to certain websites or social media outlets during school hours.

## Conclusions

In our study, a strong family environment characterized by cohesiveness, low conflict, and expressiveness, a supportive school environment, and high academic performance acted as positive influences against IA. In conclusion, minimizing adolescent Internet addiction requires a multifaceted strategy that includes both the school and family settings. The school environment may play a crucial role in teaching and creating awareness among students about the consequences of excessive Internet usage, as well as providing them with alternate activities. Family support, on the other hand, may offer adolescents a secure and caring setting in which to restrict their Internet usage and cultivate good connections and behaviors. These aspects need to be considered by policymakers and educational institutions to ensure that adolescents get the necessary help and resources to reduce addictive Internet behaviors. This might include sponsoring technological education initiatives, putting in place rules governing Internet use in classrooms, and giving support services to parents who might want extra help in supervising their adolescents’ online use. By addressing these issues, we can reduce Internet addiction among adolescents and promote healthy technology usage among the youth.

## Data Availability

The datasets generated and/or analysed during the current study are not publicly available but are available from the corresponding author on reasonable request.

## List of Abbreviations

BFRS: Brief Family Relation Scale
HBSC: Health Behaviour in School-Aged Children
IA: Internet addiction
WEIRD: Western, educated, industrialized, rich, and democratic
